# The Association between Direct Oral Anticoagulants Prescribing Behavior and Non-Valvular Atrial Fibrillation Outcomes: An Instrumental Variable Analysis of Real-World Data

**DOI:** 10.3390/jcm12227190

**Published:** 2023-11-20

**Authors:** Nipun Atreja, Stevan Geoffrey Severtson, Jenny Jiang, Chuan Gao, Dionne M. Hines, Dong Cheng, Melissa Hagan, Janis L. Breeze, Jessica K. Paulus, Eric A. Secemsky

**Affiliations:** 1 Bristol Myers Squibb, Lawrenceville, NJ 08648, USA; 2 OM1 Inc., Boston, MA 02116, USA; 3 Pfizer Inc., New York, NY 10001, USA; 4 Richard A. and Susan F. Smith Center for Outcomes Research in Cardiology, Division of Cardiovascular Medicine, Beth Israel Deaconess Medical Center, Boston, MA 02215, USA

**Keywords:** instrumental variable, rivaroxaban, apixaban, provider prescribing preference, non-valvular atrial fibrillation

## Abstract

Several observational studies have compared apixaban with rivaroxaban in patients with non-valvular atrial fibrillation (NVAF), but these analyses may be confounded by unmeasured characteristics. This study used provider prescribing preference (PPP) as an instrumental variable (IV) to assess the association between prescriber choice of rivaroxaban vs. apixaban and the study outcomes of stroke/systemic embolism (SE), major bleeding, and death in a retrospective cohort of NVAF patients in the US. Initiators of either medication were linked to their prescribers and followed until the first of the study outcome, the end of rivaroxaban/apixaban use, or 365 days after initiation. PPP for each patient was the percent of rivaroxaban initiations issued by the provider for the prior 10 NVAF patients. Cox regression models tested associations between quintiles of PPP and each outcome. A total of 61,155 patients and 1726 providers were included. The IV was a strong predictor of rivaroxaban prescription (OR = 17.9; 95% CI: 16.6, 19.3). There were statistically significant associations between increasing preference for rivaroxaban and rates of major bleeding (ptrend = 0.041) and death (ptrend = 0.031), but not stroke/SE (ptrend = 0.398). This analysis provides evidence of the relative safety of apixaban over rivaroxaban for the risk of major bleeding and death.

## 1. Introduction

Atrial fibrillation (AF) is the most common arrhythmia seen in clinical practice, affecting over 40 million patients worldwide [1,2]. AF is associated with high morbidity and mortality risk, mainly due to the elevated risk of ischemic stroke. Non-vitamin K oral anticoagulants (NOACs) are currently recommended as first-line therapy for patients with non-valvular AF (NVAF) based on evidence from large, randomized trials demonstrating the non-inferiority or superiority of factor IIa (dabigatran) and factor Xa (rivaroxaban, apixaban, and edoxaban) inhibitors versus warfarin in reducing the risk of stroke, systemic thromboembolism, and major bleeding events [3,4,5,6].

Since their introduction in 2010, the use of NOACs has greatly increased, accounting for almost 80% of incident oral anticoagulant prescriptions to NVAF patients by early 2017 [7]. The National Cardiovascular Data Registry Practice Innovation and Clinical Excellence (PINNACLE) Registry observed increasing usage of rivaroxaban and apixaban in patients with valvular AF between 2013 and 2019; overall, they jointly accounted for almost 90% of the NOACs prescribed to valvular AF patients at their most recent clinical encounter [8]. In a recent study of almost 400,000 Medicare beneficiaries diagnosed with AF and prescribed a NOAC within 12 months, apixaban was the most commonly used (77.7%), followed by rivaroxaban (16.0%) and warfarin (6.1%); dabigatran was prescribed to 0.2% of patients [9]. However, relatively limited data are available regarding head-to-head comparisons between the NOACs, and there are currently no randomized clinical trials of rivaroxaban vs. apixaban. However, evidence from several real-world data (RWD) observational studies has suggested apixaban may be safer than rivaroxaban, with reduced risks of bleeding and stroke [10,11,12,13,14,15].

However, observational studies of NOAC treatment effects may be biased due to unmeasured confounding since treatment decisions may be influenced by unknown or unmeasured variables, such as patient demographics, comorbidities, and medication pricing or availability. Instrumental variables (IVs) offer an approach to address the limitations of unmeasured confounding [16]. IVs are meant to allow for pseudo-randomization by taking advantage of natural variations in clinical practices. An IV is a variable that predicts treatment selection and is only associated with the outcome of interest via the effect on the treatment variable or via other measurable pathways. Provided the IV meets necessary assumptions, estimating the association between the IV and patient outcomes can circumvent the confounding associated with factors that affect the treatment decision for individual patients, allowing for a less biased estimate of the treatment effect. The key assumptions of an IV are not directly testable but can be considered and explored using clinical and statistical reasoning [17].

Prescribing preference-based variables offer a natural candidate for an IV as providers or facilities tend preferentially to use one NOAC over another in ways that may be less driven by individual patient characteristics that introduce confounding bias [18]. Such preference-based IVs are increasingly used to estimate causal effects in cardiovascular pharmacoepidemiology [19]. A recent observational study conducted in Denmark by Bonde and colleagues [14] applied an IV design using a facility-level preference for rivaroxaban over apixaban for stroke prevention and found an association between rivaroxaban preference and risk of major bleeding. Insight into individual physician preferences, rather than facility preferences, would capture a broader set of prescribers outside of the hospital setting while also providing control of unmeasured confounders. The current study therefore examined provider prescribing patterns as an instrument to compare outcomes (one-year rates of stroke or systemic embolism (SE), major bleeding, and all-cause mortality) in a large, US-based database of patients with NVAF.

## 2. Materials and Methods

This was a retrospective observational longitudinal cohort study of patients with NVAF initiating treatment with selected NOACs (apixaban or rivaroxaban) in the US. The study period spanned January 2013 through September 2022. Each patient’s study period began up to five years before their initial treatment of apixaban or rivaroxaban (index date), or January 2013, whichever was later; the earliest index date in the study was January 2014. Data were derived from the OM1 Real-World Data Cloud (RWDC), a multi-source, real-world proprietary data network with linked healthcare claims, social determinants, and electronic medical record (EMR) data on patients in the US [20], and a linked dataset on prescribers. All participant and provider data are fully de-identified. Patient baseline characteristics (including age, sex, race, ethnicity, insurance type, body mass index (BMI), Charlson Comorbidity Index (CCI), CHA2DS2-VASc score for atrial fibrillation stroke risk, and modified HAS-BLED score for major bleeding risk) were based on all available data within 12 months prior to or on the index date. Prescriber characteristics (years in practice, specialty, geographic region, and setting) were based on all available data prior to the index date.

Adult (≥18 years) patients with NVAF were eligible to be included if they newly initiated treatment with apixaban or rivaroxaban between January 2013 and March 2022. For patients who initiated both medications over this time period, the first treatment was included in the study, and these patients were censored on the date they initiated the other treatment. Patients with codes indicating venous thromboembolism, transient AF, cardiac surgery, rheumatic mitral valvular heart disease, or a valve replacement procedure during the 12 months prior to treatment initiation were excluded, as were those with hip/knee replacement surgery during the 6 weeks prior to treatment initiation. Patients were also excluded if they had a prescription for oral anticoagulants (OAC) during the 12 months preceding treatment initiation or prescriptions for more than 1 OAC on the index date, including apixaban, rivaroxaban, warfarin, dabigatran, and edoxaban. Eligible prescribers had a unique prescriber identification number in the OM1 RWDC and were required to prescribe at least one initial prescription for either apixaban or rivaroxaban among the eligible patients during the study period. Providers who treated fewer than 20 NVAF patients were excluded from the analysis since they would have insufficient numbers of patients to calculate provider prescribing preference (PPP) (described below).

For the two drugs of primary interest (apixaban and rivaroxaban), days’ supply of each written or filled prescription was used as a proxy for exposure time when medication discontinuation was not observed in EMR. Exposure time was censored at the earliest of the following: (1) the patient’s last EMR or claims encounter, (2) 30 days after the index treatment discontinuation date (based on days’ supply or indication of discontinuation in EMR), (3) index treatment switch date (to the other drug of interest or to warfarin, dabigatran, or edoxaban), or (4) 365 days after treatment initiation.

### 2.1. Statistical Analysis

#### 2.1.1. IV Definition

The IV used in this study was PPP for rivaroxaban over apixaban for patients with NVAF, based on the previous 10 rivaroxaban or apixaban initiators treated by the same provider. As defining the PPP is highly specific to the clinical context, clinical (EAS and MA) and epidemiological expertise (JKP and MH) were used to support the choice of primary instrument size. A threshold of 10 patients was found to result in a stronger IV and better balance of confounders across levels of the IV than thresholds of 1 or 5 in another internal medical setting [21]. Sensitivity analyses to evaluate the robustness of the results using alternative numbers of previous patients (n = 5, n = 20) to define PPP were also conducted.

The PPP was determined by ordering each provider’s patients into groups of 10 based on their index dates. For each group, the proportion of NVAF patients initiated on rivaroxaban was calculated to obtain the PPP for rivaroxaban for the next 10 patients treated by the same provider, independent of the treatment (either rivaroxaban or apixaban) actually prescribed. The proportion of patients initiated on rivaroxaban among the next 10 patients with NVAF (patients 11–20 from the same provider) was used to determine the IV for the next 10 patients with NVAF (patients 21–30 from the same provider), and so on. A random number generator was used to order patients with the same index date and provider. The number of patients in each group for a provider could range from 10 to 19. For example, if a provider prescribed treatment for 35 NVAF patients, these patients were grouped into blocks of 10, 10, and 15 based on their index dates. Finally, providers and their patients were categorized into quintiles of the PPP: 0% to 20%, 21% to 40%, 41% to 60%, 61% to 80%, and 81% to 100%.

#### 2.1.2. Evaluation of the IV

Instrument strength was described by plotting the percentage of patients initiating rivaroxaban (i.e., actual treatment received) against the percentage of the previous 10 patients from the same provider who initiated rivaroxaban (i.e., the IV). A logistic regression model, with the actual choice of the treatment for the patient as the dependent variable, was also used to describe the association with the IV, with and without measured baseline characteristics as covariates. The covariates included age, sex, race, ethnicity, insurance type, body mass index (BMI), Charlson comorbidity index (CCI), CHA2DS2-VASc score for atrial fibrillation stroke risk, and modified HAS-BLED score for major bleeding risk. As the CCI, CHA2DS2-VASc, and HAS-BLED scores are not commonly available in structured RWD, these scores are calculated based on individual patient comorbidities and demographic variables.

The IV validity assumption (i.e., that it is not associated with confounders) could not be tested directly but was assessed by qualitatively comparing the distribution of baseline patient characteristics across quintiles of the IV, similar to evaluating the balance in characteristics after randomization. To control for residual confounding, the final analyses adjusted for variables selected a priori that were prognostically meaningful (CCI, CHA2DS2-VASc score, and modified HAS-BLED score) with respect to the three study outcomes.

#### 2.1.3. Outcomes

The association between PPP and each of the following study outcomes was assessed over one year of follow-up: (1) bleeding events resulting in hospitalization while on index treatment, (2) all-cause death, and (3) stroke or SE while on index treatment or within 30 days following discontinuation of treatment. For each outcome, two Cox proportional hazards (PH) models were performed: an unadjusted model, which assumed the validity of the IV, and a model that adjusted for potential confounders under the assumption that the IV was not valid, or in other words that patient factors directly or indirectly affected the PPP. The adjusted model included CCI, CHA2DS2-VASc score, and modified HAS-BLED score, averaged (i.e., the arithmetic mean) across the previous 10 patients treated by each provider to anticipate confounding by the “case mix” of the provider. These models estimated hazard ratios (HRs) and their corresponding 95% confidence intervals (CIs) for each quintile of the IV, with “0–20% rivaroxaban preference” as the reference group. A *p*-value for trend was calculated by including the IV in its continuous instead of categorical form. The person years, number of events, and incidence rates (events per 100 person years of follow-up) for each quintile were also tabulated. The PH assumption was tested for each model by including an interaction term of the continuous IV value multiplied by the log of survival time, and the assumption of PH was not observed to be violated for the three outcomes. All analyses were performed using SAS v9.4 (SAS Institute, Cary, NC, USA), using a two-sided alpha of 0.05. This study was determined to be exempt from IRB oversight due to only de-identified participant data being used.

## 3. Results

A total of 182,071 NVAF patients initiating rivaroxaban (N = 58,604) or apixaban (N = 123,467) were identified and linked with 47,758 providers associated with the index NOAC prescription. When the IV for PPP was applied, a total of 61,155 patients (N = 16,784 prescribed rivaroxaban; N = 44,371 prescribed apixaban) associated with 1726 providers were included. Characteristics of the NVAF patients by actual treatment received are shown in Supplementary Table S1.

The distribution of patient characteristics, including age, sex, race, ethnicity, and BMI across quintiles of PPP, is shown in Table 1. Variables strongly associated with key outcomes (CCI, CHA_2_DS_2_-VASc score, and modified HAS-BLED score) were evenly distributed across PPP quintiles.

Prescriber characteristics are described in Table 2. While these data were missing for a large proportion (range 62–67%) of providers, available data showed that just over half (54.3%) of prescribers had been in practice for at least 25 years, and 67.8% specialized in cardiology, clinical cardiac electrophysiology, or interventional cardiology.

### 3.1. Evaluation of the IV

#### 3.1.1. Instrument Strength

**Figure 1:**
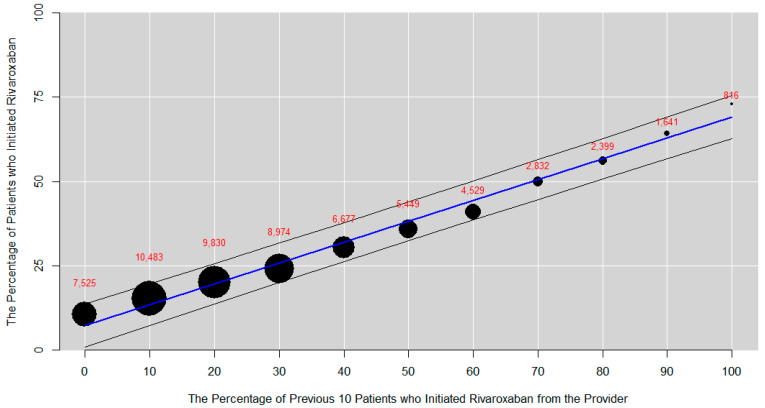
**Percent of patients initiated on rivaroxaban versus percent of previous 10 patients from a provider who initiated rivaroxaban.** The OR and 95% CI for the unadjusted and adjusted associations between the IV and actual treatment received were 17.9 (16.6–19.3) and 19.0 (17.6–20.6), respectively. The adjusted model included age, sex, race, ethnicity, insurance type, BMI, Charlson Comorbidity Index, CHA_2_DS_2_-VASc score, and modified HAS-BLED score. The size of each bubble represents the number of patients for that decile of the IV, denoted in red above.

A strong association was observed between the proportion of patients who initiated rivaroxaban (i.e., their actual prescribed treatment) and the PPP (i.e., percent of the previous ten patients who were prescribed rivaroxaban) (Figure 1). In both unadjusted and adjusted logistic regression analysis, there was a nearly 20-fold increase in the odds of a new patient being prescribed rivaroxaban for each percentage point increase in the PPP (unadjusted OR: 17.9; 95% CI: 16.6, 19.3, and adjusted OR: 19.0; 95% CI: 17.6, 20.6).

#### 3.1.2. Instrument Validity

The distributions of measured demographic variables, including age, sex, race, and ethnicity, were well balanced across levels of PPP (Table 1), including the variables known to be prognostically related (BMI, CCI, CHA_2_DS_2_-VASc score, and modified HAS-BLED score) to the outcomes of interest.

### 3.2. Primary Analyses

In the unadjusted analysis, there was a 28% greater hazard of major bleeding associated with the highest preference for rivaroxaban (81–100%), relative to the lowest (0–20%) (HR_80-100_ = 1.28; 95% CI: 0.89, 1.85, p_trend_ = 0.079 [Table 3]); however, this association did not reach statistical significance. After adjustment, the hazard increased to 34% (HR_80-100_ = 1.34; 95% CI: 0.92, 1.93), and the test for trend across the five levels of PPP was statistically significant (p_trend_ = 0.041).

The highest preference for rivaroxaban was also non-significantly associated with a higher risk of all-cause death compared to the lowest in the unadjusted (HR_80-100_ = 1.17; 95% CI: 0.56, 2.42) and adjusted (HR_80-100_ = 1.22; 95% CI: 0.58, 2.53) models. The test for trend across the five levels was statistically significant in both the unadjusted (p_trend_ = 0.042) and adjusted (p_trend_ = 0.031) models (Table 3).

There were no statistically significant associations observed in either the unadjusted (HR_80-100_ = 0.85; 95% CI: 0.44, 1.61, p_trend_ = 0.260) or adjusted (HR_80-100_ = 0.90; 95% CI: 0.47, 1.73, p_trend_ = 0.398) analyses that examined preference for rivaroxaban and the outcome of stroke/SE (Table 3).

### 3.3. Sensitivity Analyses

When the PPP was defined using the previous five patients, the sample size increased to 95,053 patients and 4866 providers (Table 4), and the instrument remained strongly associated with treatment with rivaroxaban versus apixaban (OR = 9.7; 95% CI: 9.2, 10.2). The strongest rivaroxaban preference (81–100%) was associated with a significantly higher rate of major bleeding relative to the lowest rivaroxaban preference (unadjusted HR_80-100_ = 1.35; 95% CI: 1.04, 1.76), and a stepwise increase across the five ordinal categories of the IV was also observed (p_trend_ = 0.029). No significant associations with death (p_trend_ = 0.775) or stroke/SE (p_trend_ = 0.597) were observed.

Similarly, when PPP was defined based on the prior 20 patients (Table 4), the IV was strong (OR = 23.0; 95% CI: 20.6, 25.6) but included fewer providers and patients (37,283 patients, 666 providers). The association between PPP and major bleeding was not statistically significant (HR_80-100_ = 1.26; 95% CI: 0.74, 2.14, p_trend_ = 0.570). The association between PPP and death was significant for the third and fourth quintiles of rivaroxaban preference (unadjusted HR_41-60_ = 1.93; 95% CI: 1.10, 3.38; unadjusted HR_61-80_ = 2.05; 95% CI: 1.01, 4.17), with p_trend_ = 0.012. There was no significant association with stroke/SE (p_trend_ = 0.626).

## 4. Discussion

This study used provider NOAC prescribing preference as an IV to address unmeasured confounding in the association between NOAC treatment and risk of major bleeding, death, or stroke/SE. An elevated rate of major bleeding was observed among patients treated by providers with a greater prescribing preference for rivaroxaban in the year after the index prescription, although this finding was not statistically significant in unadjusted analyses. This association was statistically significant when more patients were included (i.e., in sensitivity analysis using n = 5 patients for the IV) and after adjusting for predictors of the outcome (CCI, CHA2DS2-VASc score, and modified HAS-BLED score).

Several prior large RWD-based studies have also found a reduced risk of major bleeding for apixaban compared to other treatments. Lip and colleagues observed that those treated with apixaban had lower rates of major bleeding versus warfarin, whereas those treated with rivaroxaban had similar rates of major bleeding when compared to warfarin [15]. A meta-analysis of 10 retrospective RWD studies comparing rivaroxaban and apixaban similarly found that apixaban treatment was associated with a lower risk of major bleeding and gastrointestinal bleeding [22]. Among a population-based cohort of over 500,000 patients across four countries, Lau et al. observed that apixaban was associated with an almost 30% reduction in risk of gastrointestinal bleeding compared to rivaroxaban [23], and Ray and colleagues found that apixaban compared to rivaroxaban use was associated with a significantly increased risk of major ischemic or hemorrhagic events, among nearly 600,000 Medicare beneficiaries with AF aged 65 years or older [10]. The study by Bonde et al. used a similar analytic approach as that used in our study, with facility preference for rivaroxaban as an IV, and found that preference for rivaroxaban was associated with an elevated risk of major bleeding [14]. In light of these studies, the updated 2023 Beers Criteria from the American Geriatric Society recommend that physicians avoid rivaroxaban for long-term treatment of AF in older adults, noting the increased risk of major bleeding compared to other anticoagulants, particularly apixaban [24].

Our study also observed higher rates of all-cause mortality over one year with increased prescribing preference for rivaroxaban, an association not detected by Bonde and colleagues over two years of follow-up [14]. Given the small number of observed deaths and resulting imprecision in estimates, caution is needed in the interpretation of this finding, but it is supported by other RWD studies of NVAF patients [25]. The lack of association with stroke is consistent with Bonde, as well as the recent systemic review and meta-analysis of randomized clinical trials and real-world data that concluded that apixaban and rivaroxaban had similar efficacy for preventing stroke/SE [11].

When using an IV to assess comparative effectiveness and safety, evaluation of the key assumptions of an IV is critical. In this analysis, the IV was observed to strongly predict patient treatment and a reasonable balance of demographic and prognostic factors across levels of the IV was observed [26]. The point estimates for the association between the prescribing preference and the key outcomes were also stable across sensitivity analyses of instrument size (n = 5, n = 20). Additional adjustment for the provider case mix (i.e., average risk score of the prior set of patients) had little impact on HR point estimates, suggesting minimal impact of residual confounding. Nonetheless, the potential for residual and unmeasured confounding remains. As noted above, several studies suggest that apixaban is associated with a reduced risk of major bleeding compared to rivaroxaban. Providers who treat more AF patients may be more aware of these findings and may have changed their prescribing patterns.

### Strengths and Limitations

The OM1 RWDC features a broad representation of the US population given the number of lives covered (>310 million since 2013), which results in a geographically, ethnically, and economically diverse sample of AF patients that should well reflect patient, provider, and practice factors. Linked EMR, claim data, and other RWD sources permit deep characterization of both patients and providers. Data from over 60,000 AF patients and from over 1700 providers were analyzed in executing the objectives of this study.

In this study, days’ supply of each prescription written (EMR) or filled (claims) was used as a proxy for NOAC exposure, but whether the patient filled the prescription cannot be confirmed in EMR data, nor whether they took the medication for the duration of time as prescribed. Due to the open and multi-source nature of the OM1 RWDC, there is some possibility that patients may have continued treatment but were lost to follow-up if they received their prescriptions from another provider. In addition, most patients were treated by providers who treated fewer than 20 NVAF patients who initiated rivaroxaban or apixaban during the study period; this restriction resulted in nearly two-thirds of patients being excluded from the primary analysis cohort. The loss of patients due to both provider size and loss of follow-up reduced the statistical power of the study and may have resulted in a less representative sample of patients.

## 5. Conclusions

An association between prescribing preference for rivaroxaban and increased rate of major bleeding was observed across all analyses and was statistically significant in adjusted analysis and in sensitivity analysis with increased sample size. An association between prescribing preference for rivaroxaban and risk of all-cause death was also observed, but not between increased rivaroxaban preference and rate of stroke/SE. Prescriber preference for NOACs offers promise to provide insight into the association between the choice of these therapies and the risk of major bleeding and other outcomes. These results provide evidence for the relative safety of apixaban over rivaroxaban for bleeding risks and death in this US population.

## Figures and Tables

**Table 1:** Baseline demographic and clinical characteristics of patients (N = 61,155), by quintile of the instrumental variable.

	Percent of Provider’s Previous 10 Patients with NVAF Initiated on Rivaroxaban *				
	0–20% (N = 27,838)	21–40% (N = 15,651)	41–60% (N = 9978)	61–80% (N = 5231)	81–100% (N = 2457)
Mean age (SD)	72.6 (10.0)	72.5 (9.9)	72.9 (9.7)	72.2 (9.9)	72.1 (9.7)
Received rivaroxaban	4361 (15.7%)	4203 (26.9%)	3812 (38.2%)	2759 (52.7%)	1649 (67.1%)
**Sex**					
Female	11,958 (43.0%)	6793 (43.4%)	4219 (42.3%)	2188 (41.8%)	1024 (41.7%)
Male	15,880 (57.0%)	8858 (56.6%)	5759 (57.7%)	3043 (58.2%)	1433 (58.3%)
**Race**					
Black	1647 (6.9%)	895 (6.7%)	405 (4.8%)	216 (4.9%)	140 (6.8%)
White	21,845 (92.1%)	12,318 (92.3%)	7855 (94.0%)	4105 (93.9%)	1886 (92.1%)
Other	239 (1.0%)	126 (0.9%)	93 (1.1%)	52 (1.2%)	21 (1.0%)
Unknown	4107	2312	1625	858	410
**Ethnicity**					
Hispanic	807 (3.7%)	384 (3.1%)	244 (3.2%)	112 (2.8%)	45 (2.5%)
Not Hispanic	21,166 (96.3%)	11,835 (96.9%)	7337 (96.8%)	3874 (97.2%)	1773 (97.5%)
Unknown	5865	3432	2397	1245	639
**Insurance type**					
Commercial	2024 (7.5%)	1085 (7.1%)	741 (7.6%)	341 (6.7%)	195 (8.1%)
Medicaid	97 (0.4%)	56 (0.4%)	37 (0.4%)	20 (0.4%)	4 (0.2%)
Medicare	9179 (34.0%)	5311 (34.7%)	3441 (35.4%)	1801 (35.3%)	885 (36.8%)
Multiple	6860 (25.4%)	3746 (24.5%)	2322 (23.9%)	1108 (21.7%)	519 (21.6%)
Other	8873 (32.8%)	5104 (33.4%)	3193 (32.8%)	1830 (35.9%)	803 (33.4%)
Unknown	805	349	244	131	51
Mean body mass index (SD)	30.5 (6.9)	30.6 (6.9)	30.4 (6.8)	30.6 (6.8)	30.8 (7.0)
Median Charlson Comorbidity Index (IQR)	0 (0–2)	0 (0–2)	0 (0–2)	0 (0–2)	0 (0–2)
Median CHA_2_DS_2_-VASc score (IQR)	3 (2–5)	3 (2–5)	3 (2–5)	3 (2–4)	3 (2–4)
Median Modified HAS-BLED score (IQR)	2 (2–3)	2 (2–3)	2 (2–3)	2 (2–3)	2 (1–3)

Abbreviations: IQR, interquartile range; NVAF, non-valvular atrial fibrillation; SD, standard deviation; * Sample size for each quintile represents the number of patients in the cohort whose provider prescribed rivaroxaban for that percentage range of the previous 10 patients with NVAF (e.g., 27,838 patients had a provider who prescribed rivaroxaban for 0–20% of the previous 10 patients with NVAF).

**Table 2:** Provider Characteristics.

		Provider Cohort (N = 1724)
Years in practice	0–2	3 (0.5%)
	3–5	8 (1.4%)
	6–9	23 (4.1%)
	10–14	52 (9.3%)
	15–19	90 (16.0%)
	20–24	81 (14.4%)
	≥25	305 (54.3%)
	Unknown	1162

Specialty	Cardiology	336 (51.1%)
	Clinical Cardiac Electrophysiology	66 (10.0%)
	Internal Medicine	159 (24.2%)
	Interventional Cardiology	44 (6.7%)
	Other	53 (8.1%)
	Unknown	1066

Geographic Region	Midwest	92 (14.0%)
	Northeast	77 (11.7%)
	South	409 (62.2%)
	West	80 (12.2%)
	Unknown	1066

Setting	Academic	12 (1.8%)
	Community	80 (12.3%)
	Both	560 (85.9%)
	Unknown	1072

**Table 3:** Unadjusted and adjusted hazard ratios for outcomes at 365 days by quintile of the instrumental variable.

Outcome	Percent of Previous 10 Patients with NVAF from Provider Initiated on Rivaroxaban	Number of Patients with Events	Person Years	Incidence Rate per 100 Person Years	Unadjusted HR (95% CI)	*p*-Value for Trend	Adjusted HR * (95% CI)	*p*-Value for Trend
Major Bleeding	0–20%	257	11,100.68	2.315	Reference	0.079	Reference	0.041
	21–40%	151	6648.11	2.271	0.99 (0.81, 1.21)		0.99 (0.81–1.22)	
	41–60%	104	4339.95	2.396	1.04 (0.83, 1.31)		1.06 (0.85–1.34)	
	61–80%	61	2266.63	2.691	1.17 (0.89, 1.55)		1.21 (0.91–1.60)	
	81–100%	32	1090.48	2.934	1.28 (0.89, 1.85)		1.34 (0.92–1.93)	
Death	0–20%	72	11,163.10	0.645	Reference	0.042	Reference	0.031
	21–40%	45	6688.04	0.673	1.06 (0.73, 1.54)		1.07 (0.74–1.55)	
	41–60%	39	4364.34	0.894	1.43 (0.97, 2.11)		1.44 (0.97–2.13)	
	61–80%	20	2281.26	0.877	1.40 (0.85, 2.29)		1.43 (0.87–2.36)	
	81–100%	8	1099.95	0.727	1.17 (0.56, 2.42)		1.22 (0.58–2.53)	
Stroke/Systemic Embolism	0–20%	121	11,133.18	1.087	Reference	0.260	Reference	0.398
	21–40%	57	6670.06	0.854	0.79 (0.58, 1.08)		0.80 (0.58–1.10)	
	41–60%	40	4352.03	0.919	0.85 (0.60, 1.22)		0.88 (0.61–1.25)	
	61–80%	17	2276.64	0.747	0.69 (0.42, 1.15)		0.73 (0.44–1.21)	
	81–100%	10	1098.08	0.911	0.85 (0.44, 1.61)		0.90 (0.47–1.73)	

Abbreviations: CI, confidence interval; HR, hazard ratio; NVAF, non-valvular atrial fibrillation; * Adjusted for average Charlson Comorbidity Index, average CHA_2_DS_2_-VASc score, and average modified HAS-BLED score of the previous 10 patients treated by each provider.

**Table 4:** Sensitivity analyses: Unadjusted hazard ratios for outcomes at 365 days using different definitions of the instrumental variable.

Outcome	Percent of Previous N Patients with AF from Provider Initiated on Rivaroxaban	IV Variable Based on Previous 5 Patients	IV Variable Based on Previous 20 Patients
		N = 95,053 Patients; 4866 Providers	N = 37,283 Patients; 666 Providers
Major Bleeding	0–20%	Reference	Reference
	21–40%	1.05 (0.89, 1.23)	0.96 (0.75, 1.24)
	41–60%	1.04 (0.86, 1.25)	0.98 (0.73, 1.31)
	61–80%	1.20 (0.97, 1.48)	1.09 (0.75, 1.60)
	81–100%	1.35 (1.04, 1.76)	1.26 (0.74, 2.14)
	*p*-value for trend	0.029	0.570
Death	0–20%	Reference	Reference
	21–40%	0.98 (0.75, 1.27)	1.47 (0.87, 2.50)
	41–60%	1.09 (0.81, 1.46)	1.93 (1.10, 3.38)
	61–80%	0.86 (0.59, 1.27)	2.05 (1.01, 4.17)
	81–100%	0.98 (0.61, 1.60)	1.89 (0.66, 5.44)
	*p*-value for trend	0.775	0.012
Stroke/Systemic Embolism	0–20%	Reference	Reference
	21–40%	0.86 (0.66, 1.11)	0.94 (0.64, 1.38)
	41–60%	0.97 (0.73, 1.29)	0.87 (0.55, 1.37)
	61–80%	1.14 (0.82, 1.57)	1.05 (0.59, 1.88)
	81–100%	1.05 (0.68, 1.63)	0.57 (0.18, 1.81)
	*p*-value for trend	0.597	0.626

Abbreviations: CI, confidence interval; HR, hazard ratio; IV: instrumental variable; NVAF, non-valvular atrial fibrillation.

## Data Availability

Restrictions apply to the availability of these data. The datasets generated and/or analyzed for the current study are not publicly available due to the confidential and proprietary nature of the datasets.

## Supplementary Materials

The following supporting information can be downloaded at: https://www.mdpi.com/article/10.3390/jcm12227190/s1. Table S1: Baseline demographic and clinical characteristics of patients (N = 61,155), by actual treatment received.
