# Supplementary material for: The Association between Direct Oral Anticoagulants Prescribing Behavior and Non-Valvular Atrial Fibrillation Outcomes: An Instrumental Variable Analysis of Real-World Data
Source: J Clin Med. 2023 Nov 20;12(22):7190. doi: 10.3390/jcm12227190 (PMC10671855; doi:10.3390/jcm12227190)
Supplement: Supplementary file 1 [file jcm-12-07190-s001.zip › jcm-2673286-supplementary.pdf]

Table S1. Baseline demographic and clinical characteristics of patients (N = 61,155), by actual treatment received.

	Actual Treatment Received		
	**Apixaban (N = 44,371)**	**Rivaroxaban (N = 16,784)**	**Total (N = 61,155)**
Mean age (SD)	73.2 (9.8)	71.1 (10.2)	72.6 (9.9)
**Sex**			
Female	19,578 (44.1%)	6604 (39.3%)	26,182 (42.8%)
Male	24,793 (55.9%)	10,180 (60.7%)	34,973 (57.2%)
**Race**			
Black	2454 (5.5%)	849 (5.1%)	3303 (5.4%)
White	34,880 (78.6%)	13,129 (78.2%)	48,009 (78.5%)
Other	394 (0.9%)	137 (0.8%)	531 (0.9%)
Unknown	6643 (15.0%)	2669 (15.9%)	9312 (15.2%)
**Ethnicity**			
Hispanic	1167 (2.6%)	425 (2.5%)	1592 (2.6%)
Not Hispanic	33,592 (75.7%)	12,393 (73.8%)	45,985 (75.2%)
Unknown	9612 (21.7%)	3966 (23.6%)	13,578 (22.2%)
**Insurance Type**			
Commercial	3108 (7.0%)	1278 (7.6%)	4386 (7.2%)
Medicaid	145 (0.3%)	69 (0.4%)	214 (0.3%)
Medicare	15,152 (34.1%)	5465 (32.6%)	20,617 (33.7%)
Multiple	10,750 (24.2%)	3805 (22.7%)	14,555 (23.8%)
Other	14,044 (31.7%)	5759 (34.3%)	19,803 (32.4%)
Unknown	1172 (2.6%)	408 (2.4%)	1580 (2.6%)
Mean body mass index (SD)	30.3 (6.9)	31.1 (7.0)	30.5 (6.9)
Median Charlson Comorbidity Index (IQR)	0 (0–2)	0 (0–2)	0 (0–2)
Median CHA_2DS_2-VASc score (IQR)	4 (2–5)	3 (2–4)	3 (2–5)
Median Modified HAS-BLED score (IQR)	2 (2–3)	2 (1–3)	2 (2–3)

Abbreviations: IQR, interquartile range; SD, standard deviation
